# Long-term monitoring of sand fly populations: temporal responses to land-use change and climatic variability

**DOI:** 10.1186/s13071-026-07475-1

**Published:** 2026-06-05

**Authors:** Yoni Waitz, Shlomit Paz, Shirly Elbaz, Debora Diaz, Ira Ben-Avi, Liora Studentsky, Eran Hacklai, Shai Reicher, Ron Drori, Maia Davidovich-Cohen, Laor Orshan, Oscar David Kirstein

**Affiliations:** 1 School of Environmental Sciences, University of Haifa, Haifa, Israel; 2 Public Health Laboratories, Public Health Services (PHS), Ministry of Health (MOH), Jerusalem (PHL-J), Israel; 3 Division of Pest Control and Pesticides, Ministry of Environmental Protection, Jerusalem, Israel; 4 Israel Meteorological Service, Beit Dagan, Israel

**Keywords:** Sand flies, *Phlebotomus*, Long-term surveillance, Land-use change, Anthropogenic environmental change, Climatic variability, Population dynamics, Leishmaniasis vectors, Seasonality

## Abstract

**Background:**

Phlebotomine sand flies are the sole vector of leishmaniasis and other sand fly–borne diseases, and their populations are shaped by climate and human-driven environmental change. While warming is expected to expand sand fly ranges, the joint effects of long-term climatic variability and land-use change on population dynamics remain poorly understood, largely owing to limited long-term monitoring.

**Methods:**

We analyzed an 18-year dataset (2005–2021) from a site in semi-arid Ma’ale Adumim, Israel, using CO_2_-baited traps. Land-cover change was quantified using remote sensing, focusing on the establishment of a managed urban park near the site. Climatic variables from the Israel Meteorological Service and ERA5 were related to interannual variation in abundance and seasonal phenology characters (median seasonal timing and peak timing).

**Results:**

A marked land-cover transformation associated with an irrigated park was identified, increasing dry-season vegetation. Sand fly species showed contrasting long-term trends and phase-dependent responses: *Phlebotomus sergenti* and *P. papatasi* were most abundant immediately after landscape modification and declined later, whereas *P. tobbi* and *P. syriacus* increased after park establishment. Seasonal activity was strongly species-specific with limited interspecific synchrony. Climatic variability explained little interannual variation overall; only *P. tobbi* maximum abundance and timing of within-season population increase were associated with the timing of thermal onset.

**Conclusions:**

Anthropogenic land-use change dominated long-term sand fly population dynamics, often exceeding climatic effects, and altered habitat suitability in species-specific, phase-dependent ways. Integrating land management and spatial planning into prevention strategies is critical for reducing leishmaniasis risk in human-modified landscapes.

**Graphical Abstract:**

**Figure:**
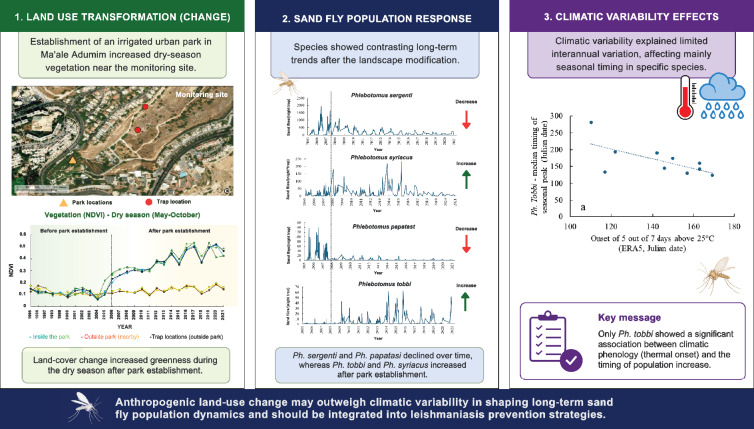

**Supplementary Information:**

The online version contains supplementary material available at 10.1186/s13071-026-07475-1.

## Background

Sand fly-borne diseases (SFBDs) include leishmaniasis, one of the most widespread vector-borne diseases worldwide, and sand fly–borne phleboviruses (SFBV) [1], are transmitted by phlebotomine sand flies of the genus *Phlebotomus* in the Old World and by *Lutzomyia* in the New World and are associated with a diverse range of reservoir hosts, including humans, wildlife, and domestic animals. Lishmaniasis is endemic in more than 90 countries and causes about 0.9–1.6 million new human cases and 20,000–50,000 deaths reported annually [2, 3]. As a neglected tropical disease, its true burden is substantially underestimated, owing to underreporting and the concentration of most cases in socioeconomically vulnerable regions [4–6].

Across the Mediterranean Basin and southern Europe, more than ten sand fly species are considered potential vectors of different *Leishmania* species [7]. The eastern Mediterranean, being relatively hot and arid and serving as an intercontinental bridge between Western Asia, Africa, and Europe, hosts a particularly rich sand fly fauna. It also exhibits some of the highest incidence rates of cutaneous leishmaniasis (CL) in the region, predominantly caused by *Leishmania major* and *Leishmania tropica*, and, to a lesser extent, *Leishmania infantum*, which is responsible for visceral leishmaniasis (VL) [8–10]. Since sand flies are ectothermic organisms and therefore temperature-dependent in their physiology and activity, their distribution is largely restricted to relatively warm regions [11, 12]. It is therefore hypothesized that, under ongoing climate change and global temperature rise, sand fly ranges will expand into higher and colder latitudes and altitudes, increasing the geographical extent of climatically suitable habitat [5, 13, 14].

The environmental characteristics of sand fly habitats are diverse, and several species show a marked preference for human-modified environments, including agricultural areas, disturbed landscapes, and peri-urban zones. In addition to climate change, land-use transformations can modify local habitat suitability and microclimatic conditions, thereby influencing sand fly abundance and species composition at the local scale. In combination with shifts in changes in climatic suitability, for example, through temperature increases, such changes may also facilitate the establishment of sand flies in newly suitable areas [15–19].

Previous studies examining the seasonal dynamics of sand fly populations have identified strong associations between temperature during sand fly activity and population size and highlighted the importance of temperature thresholds determining the seasonal onset of sand fly activity [21, 22]. Warmer early-season conditions often advance the onset of population growth. In addition, multiple seasonal patterns have been documented, ranging from unimodal summer peaks to bi- or trimodal dynamics that may be influenced by environmental factors (such as land use type) and climatic drivers (such as temperature and humidity) [22–26].

However, most studies are based on short time series and generally include only a limited number of sampling events per season. Such constraints substantially limit the ability to characterize the complex relationships between sand fly population dynamics, climatic conditions (including temperature and relative humidity), environmental characteristics such as land cover, and human-driven factors as land-use and land-management activities [15, 17, 27]. These limitations also restrict the capacity to understand how these dynamics relate to infection rates, vector infection prevalence, and disease incidence in animals and humans. Moreover, studies that explicitly investigate potential interactions between climatic variability and land-use patterns in shaping sand fly dynamics remain scarce [28].

Here, we present a long-term time-series analysis based on an 18-year sand fly monitoring dataset from a sentinel representative site, among the longest continuous datasets of its kind reported from the region, as part of the national vector surveillance program led by the Medical Entomology Laboratory (Ministry of Health) and the Ministry of Environmental Protection of Israel. This study aimed to examine seasonal and interannual sand fly dynamics in relation to key climatic drivers, and to assess how a documented land-cover transformation near the site, together with climatic variability, shapes seasonal patterns and the relative abundance of different sand fly species.

## Methods

### Study site

The Ma’ale Adumim area is a long-established focus of CL caused by *L. tropica*, with *Phlebotomus sergenti* (Parrot, 1917) recognized as the primary vector [29] and the rock hyrax (*Procavia capensis*) identified as the reservoir host [30]. The sampling area is a peri-urban site located in the Judean Desert fringe in the eastern Mediterranean region, along a semi-arid ecotone between the Mediterranean and arid climatic zones. Summers are warm and dry, ranging from May to October, with mean daily temperatures above 30 °C, and annual precipitation ranges between 150 and 250 mm [31]. The landscape is mountainous and rocky, with geological formations that include layers of chalk and flint, and is characterized by sparse herbaceous vegetation.

Over the past several decades, the area has experienced rapid peri-urban development, characterized by construction along hill ridges bordering open landscapes. Associated land modification included extensive clearing and deposition of rock debris along development margins, creating human-modified habitats with potential relevance for sand fly vectors and reservoir hosts of *L. tropica* [30, 32, 33].

### Sand fly monitoring

**Fig. 1:**
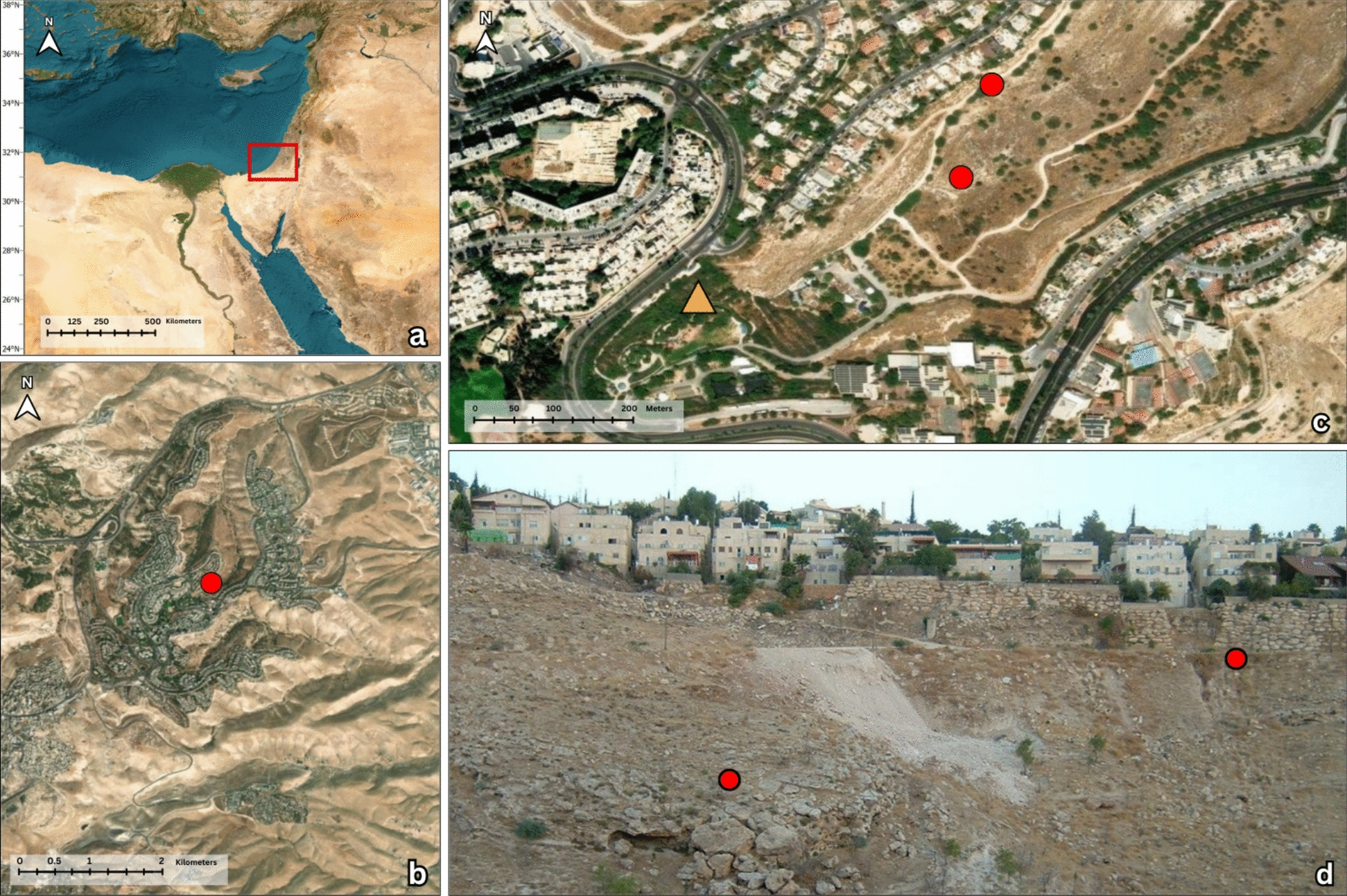
Map of the long-term monitoring location in Ma’ale Adumim. Trap locations (in red): **a** Regional map of the study site within the east Mediterranean region; **b** Orthophoto of the town Ma’ale Adumin and its environment; **c** Higher-resolution view of the trap locations and park location in orange triangle; **d** Photograph and locations of the trapping points (facing north). Photo courtesy of Dr. Laor Orshan and the IMS, unknown date

Monitoring was conducted at a fixed sentinel peri-urban site (31.779°N, 35.304°E, Fig. 1) as part of routine national surveillance led by the Medical Entomology Laboratory of the Ministry of Health, in coordination with the Ministry of Environmental Protection. Sand flies were collected using two modified Center for Disease Control (CDC)-type traps baited with CO_2_, deployed at two fixed sampling points approximately 50–100 m apart, in an updraft position with trap openings set at approximately 1 m above ground level, and operated according to standard protocols routinely used by the Medical Entomology Laboratory [33, 34].

Trapping was conducted at variable frequency (1–4 sampling events per month), primarily during the sand fly activity season (April–November), with occasional sampling during December–March in some years. Collected sand flies were transported to the laboratory, counted, sexed, and identified to species level using standard morphological keys [35].

### Sand fly data aggregation and preprocessing

For this study, monitoring data collected between 2005 and 2021 were compiled for analysis. To reduce stochastic variation and localized trap effects, catches from the two fixed trapping sites were combined for each sampling event. For subsequent analyses, the maximum trap catch per sampling date, was used as the primary measure of sand fly abundance, defined as the higher of the two trap catches when both traps were available, or the single trap catch when only one trap operated. This metric was selected over an average to reduce sensitivity to missing data and inter-trap variability while maintaining temporal resolution. Species composition among females was inferred from the proportional representation of species in male catches from the same sampling event.

### Temporal variability of populations characters

Temporal variability in sand fly populations was assessed through inter-annual comparisons focusing on abundance and seasonal timing. Annual maximal abundance for each species was defined as the mean of the three highest trap counts (sand flies per trap-night) recorded within a given season.

Seasonal phenology was characterized using multi-year abundance data, with two complementary metrics calculated for each species and year: (i) median timing, defined as the Julian day on which cumulative abundance exceeded the median value of the seasonal peak; and (ii) maximum abundance timing, defined as the Julian day corresponding to the highest recorded abundance within the season.

To ensure robustness, phenological metrics were calculated only for the year with at least six positive observations (sand flies per trap-night > 0) from the beginning of the season through October; years not meeting this criterion were excluded. Following this filtering, the final dataset used for phonological analyses spanned 2005 to 2014. Inter-annual synchrony in seasonal timing among species was assessed using pairwise Spearman rank correlations, evaluated separately for median timing and maximum abundance timing.

### Land-cover transformation detection using remote sensing analysis:

During the early years of the sand fly monitoring period, substantial landscape modification occurred in the vicinity of the study site. An intensively managed and irrigated urban park was established approximately 200–300 m southwest of the monitoring area, covering ~ 5 ha and maintaining dense perennial vegetation through year-round irrigation, including the dry summer months.

To characterize land cover changes associated with park establishment and maintenance, remote sensing analysis was conducted using Landsat 5 and Landsat 7 imagery at 30 m spatial resolution on the basis of data acquired between 1995 and 2021. A fixed set of seven sampling locations were manually selected, four within and three in the nonplanted natural landscape, to represent areas of persistent vegetation cover and used consistently across all years. Selection avoided park edges and mixed land-cover pixels. For each location, a time series of the normalized difference vegetation index (NDVI) was extracted using a point-based approach using the Microsoft Planetary Computer STAC API with one cloud-free image, acquired annually in July, corresponding to the mid-dry season [36, 37]. July imagery was selected to minimize the contribution of seasonal herbaceous vegetation and emphasize perennial and irrigated vegetation cover.

### Climate data

Climatic data for temperature were obtained from two sources. First, observations were retrieved from the formal meteorological station of the Israel Meteorological Service (IMS) located in Ma’ale Adumim [38], available from 2007 onward. Second, ERA5 reanalysis data were used to provide continuous gridded climatic information for the full study period, as IMS data were unavailable for the early years of sand fly sampling. Given known biases and limited accuracy in ERA5 estimates of local-scale precipitation and relative humidity, these variables were obtained exclusively from IMS ground-based observations [39-41].

Daily temperature variability was based on a single observation per day at 18:00, corresponding to the evening hours during which sand flies are active [20]. Precipitation was aggregated from daily measurements (≥ 1 mm) to monthly totals and to the hydrological season, defined as the rainy season from November to March. Relative humidity data were available only from IMS.

For analyses of maximal abundance, which covered the period 2007–2021, IMS data were used as the climatic data source. For phenological analyses, including median timing and timing of maximal abundance. The analysis was conducted for the period 2005–2014, which required climatic data from 2005 to 2006; therefore, ERA5 data were used for these analyses.

Climatic effects on maximal abundance and phenological metrics were evaluated using a set of derived climatic variables. Definitions, temporal aggregations, and data sources for these variables are presented in Table 1.

**Table 1:** Climatic variables and temporal aggregation used in analyses

Climatic variable	Temporal window	Definition	Data source/analysis
Mean temperature	Apr–Oct; Jun–Sep	Mean daily temperature at 18:00	IMS (maximal abundance); ERA5 (phenology)
Heat days	Apr–Oct; Apr–Jun	Number of days with temperature > 30 °C	IMS; ERA5
Onset temperature	Seasonal	Timing when temperature exceeds 20, 25, 27, 28, or 30 °C using (i) 7-day mean above threshold or (ii) ≥ 5 of 7 consecutive days above threshold	IMS; ERA5
Relative humidity	Apr–Oct; Jun–Sep	Mean relative humidity	IMS only
Precipitation	Nov–Mar; Mar–Jun	Total seasonal and late-season precipitation (mm); daily ≥ 1 mm	IMS only

IMS-derived variables were used for maximal abundance analyses (2007–2021); ERA5-derived variables were used for phenological analyses (2005–2014). Relative humidity was available from IMS only

To assess consistency between data sources, a comparison between IMS and ERA5 temperature data are provided in the Additional file (Figs. S1–S5; Tables S1–S2). Daily and monthly temperatures from the two sources showed strong correlations, with systematically higher values for IMS. Monthly average differences between IMS and ERA5 ranged from 3 to 5 °C during spring (March–April) and early winter (November–December), and from 1 to 3 °C during summer months (Fig. S4). Because of this difference, onset temperature metrics derived from the two datasets were strongly correlated when thresholds differing by approximately 5 °C were compared, indicating that the difference between the datasets is consistent across years. Given that consistency and the strong correlation between the datasets, this difference is not expected to affect the interpretation of the relationships.

### Statistical modelling

Relationships between sand fly population metrics and climatic variables were evaluated using Theil-Sen regression, a nonparametric estimator appropriate for small sample sizes and robust to outliers. Statistical significance was assessed using Kendall’s rank correlation coefficient (τ).

Owing to collinearity among climatic variables and the limited number of annual observations (up to 18 years), only one climatic variable was included in each model. Interannual variability in sand fly populations was analyzed using IMS data for maximal abundance and ERA5 data for phenological metrics, as described above. Additionally, we tested the potential effect of sampling effort, namely the number of observations per season, on the sand-fly (additional file Table S3). All statistical analyses were conducted in the R programming environment, version 4.4.1, using the *trend* and *mblm* packages. Maps were created using ArcGIS Pro (ESRI, version 3.6.0).

## Results

### Land-cover transformation

**Fig. 2:**
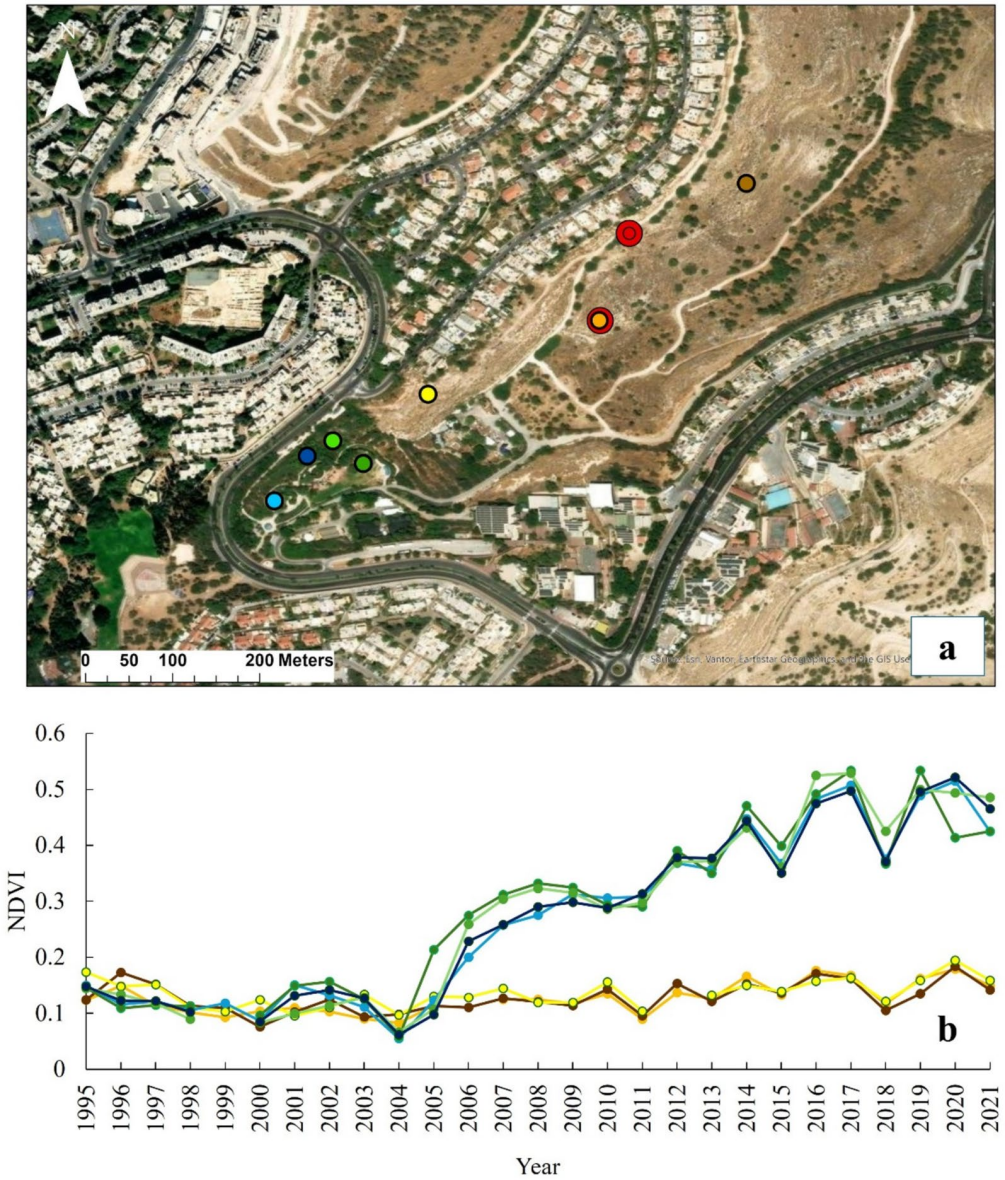
NDVI time-series for selected locations in the park and nearby. **a** Map of the selected locations for greenness cover analysis of the dry season (July) inside and outside the park, next to the traps locations (in red). Colors of the locations match the time series. **b** Time-series of greenness levels (NDVI) for seven selected locations from 1995–2021. Colors match the locations in the map

Remote sensing analysis indicated a positive change in the greenness coverage in the park area during 2005–2006, followed by a slower increase thereafter and an apparent saturation around 2018. By contrast, no significant change in vegetation cover was detected in the adjacent open area during the same period (Fig. 2). According to field records, park construction and landscaping were completed before the summer of 2007 (personal communication). This documented land-cover transformation provided the context for assessing long-term changes in sand fly populations and species composition.

### Sand fly populations and species composition

Analyses focused on the four dominant sand fly species captured during the study period: *Phlebotomus sergenti*, *Phlebotomus papatasi*, *Phlebotomus tobbi*, and *Phlebotomus syriacus*. Species captured at very low frequencies were excluded owing to insufficient sample sizes for time-series analysis.

**Fig. 3:**
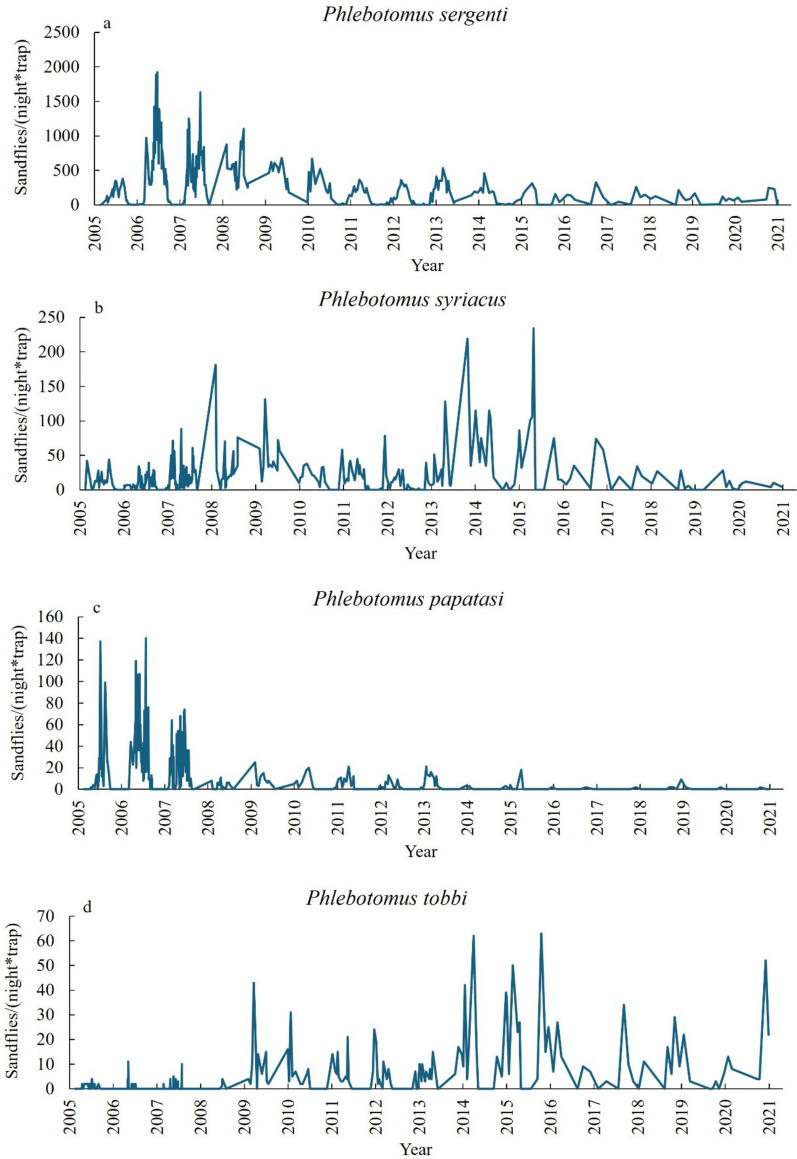
Time-series of the sand-flies species in Ma’ale-adumim, 2005–2021

A total of 199,528 sand flies were collected during the monitoring period (103,664 males and 95,864 females). The majority of captures were *Ph. sergenti* (91.3%; 182,101 individuals), followed by *Ph. syriacus* (4.9%; 9819 individuals), *Ph. papatasi* (2.9%; 5898 individuals), and *Ph. tobbi* (0.9%; 1704 individuals). Time series of maximum trap catch per sampling date for each species are shown in Fig. 3. No consistent effect was found for the sampling effort on the maximal abundance or on the phenological variables (Additional file Table S3).

### Species-specific abundance patterns

*Phlebotomus sergenti*: the highest maximum trap catch was recorded in August 2006, with 1924 individuals per trap-night. Excluding the first sampling year (2005), annual maxima showed a rapid decline after 2006, followed by a more gradual decrease, reaching values of approximately 100–200 individuals per seasonal maximum in later years.

*Phlebotomus syriacus*: the highest maximum trap catch was recorded in November 2015 with 234 individuals per trap-night. The annual maxima did not exhibit a clear long-term directional trend but showed two distinct periods of high-yield years: 2007–2009 and 2013–2015.

*Phlebotomus papatasi*: the highest maximum trap catch, 140 individuals per trap-night, occurred in October 2006. Annual maxima declined sharply after 2007, from values exceeding 70 individuals per trap-night during 2005–2007 to values below 20 in subsequent years, approaching zero toward the end of the monitoring period.

*Phlebotomus tobbi*: the highest maximum trap catch was recorded in May 2016 with 63 individuals per trap-night. Annual maxima were low during 2005–2008 (< 7 individuals per trap-night), followed by a gradual increase and a peak during 2014–2016.

### Climatic effects on maximal abundance

**Fig. 4:**
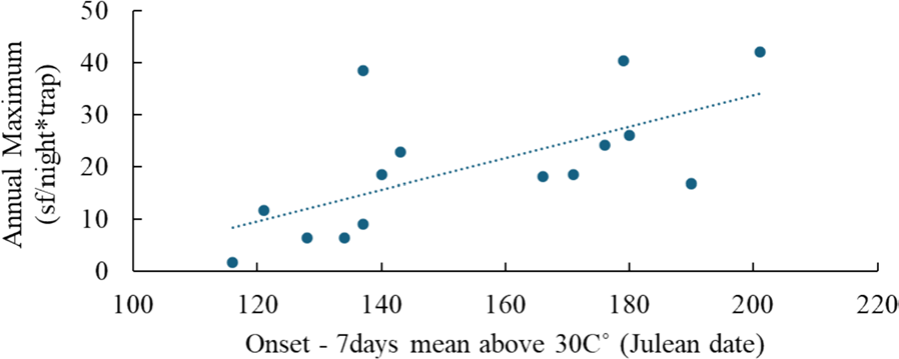
Annual maximum abundance (average of 3 trapping dates) of *Ph. tobbi* for the years 2007–2021 versus the onset of 7 days mean above 30°C

Among all models comparing relationships between climatic variables and species-specific maximal abundance. Only *Ph. tobbi,* showed a significant positive linear correlation with the timing of the hot-season onset from 2007 (after the establishment of the park) to 2021, defined as the onset of 7 days with mean temperature above 30 °C (*n *= 15, Theil–Sen slope = 0.35, Kendall’s τ = 0.57, *p* = 0.002; pseudo-R^2^ ≈ 0.34; Fig. 4).

The onset of the hot season ranged from day 116 (started on April 25, 2008) to day 202 (from July 20, 2015), indicating that a later onset of high-temperature conditions was associated with higher maximal abundance of *Ph. tobbi*. A similar but weaker association was observed when hot-season onset was defined as 5 out of 7 days above 30 °C (*n* = 15, Kendall’s τ = 0.39, *p* = 0.046; pseudo-R^2^ ≈ 0.15). Results of all tested models are provided in the additional file, Table S4.

### Phenology and inter-species synchrony

**Fig. 5:**
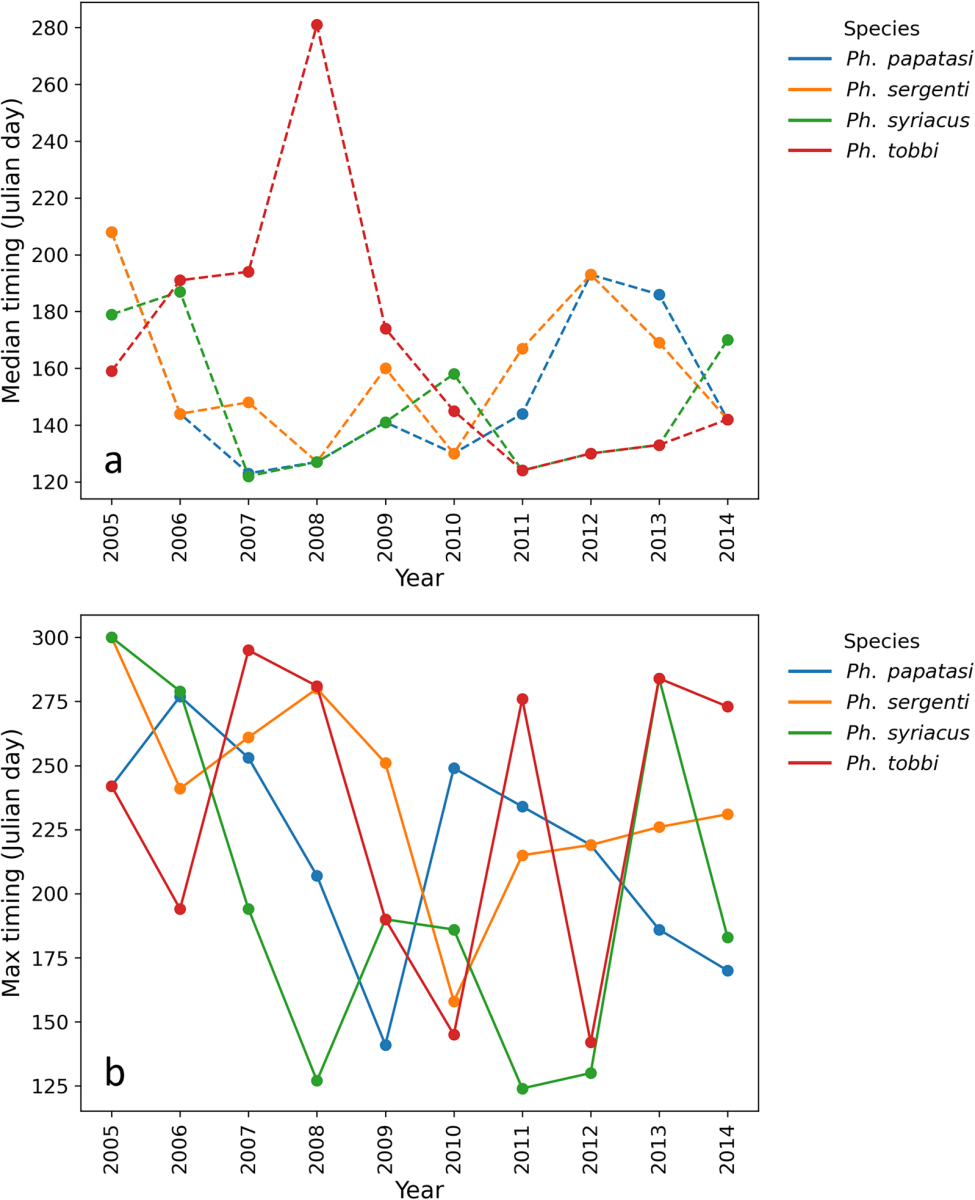
Temporal patterns in the seasonal timing of the four *Phlebotomus* species are shown for the period 2005–2014; **a** the median seasonal timing, defined as the date at which the seasonal activity exceeded the median value of the specific seasonal observations; **b** the timing of the maximum seasonal activity per year

Significant association in median seasonal timing was detected only between *Ph. sergenti* and *Ph. papatasi* (ρ = 0.80, *p* = 0.005, *n* = 10). Interannual patterns of median and maximum seasonal timing for all four species during 2005–2014 are shown in Figs. 5a and b, respectively.

**Fig. 6:**
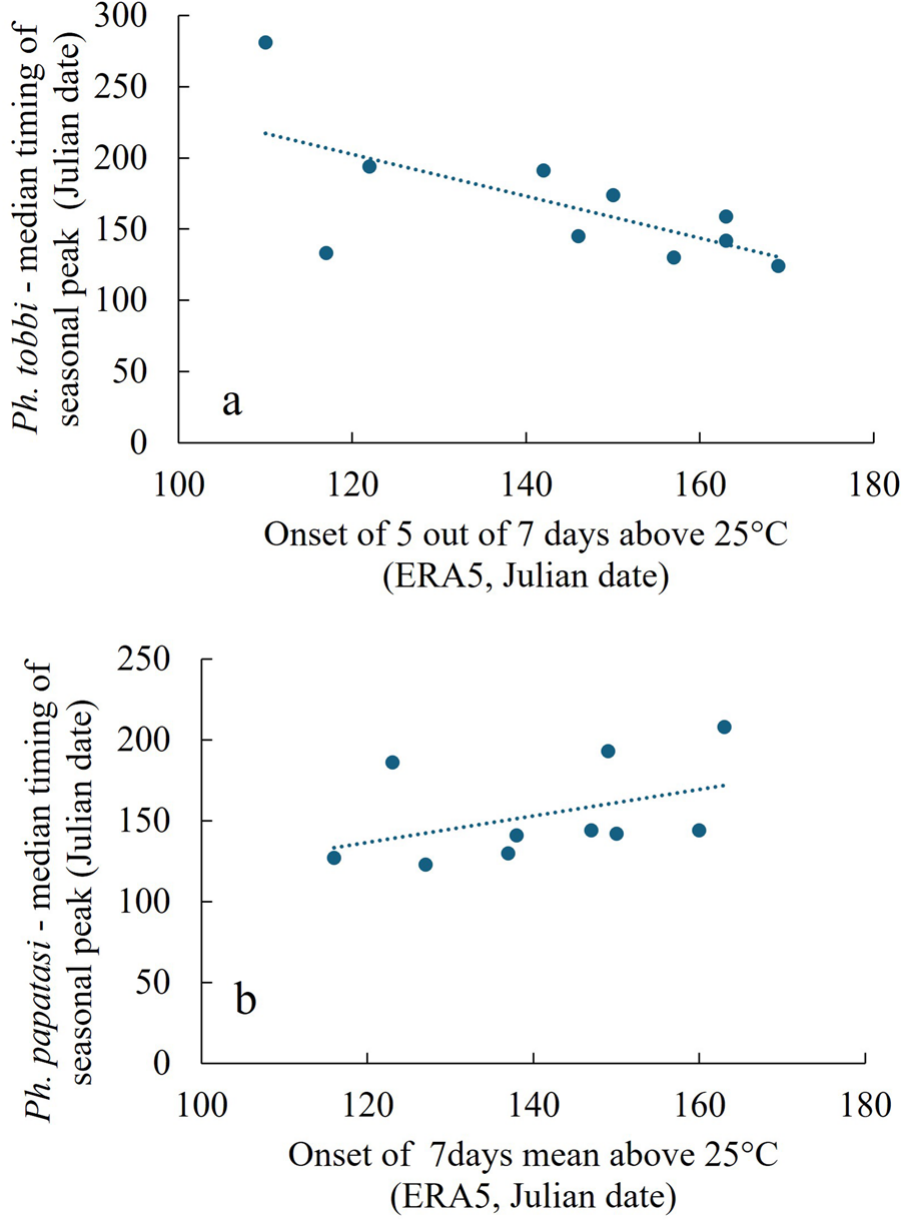
Relationships between the median seasonal timing and the thermal onset metrics for the two *Phlebotomus* species exhibiting significant climate-phenology associations with temperature onsets from ERA5 dataset; **a** *Ph. tobbi*, median seasonal versus the onset defined as the first occurrence of 5 out of 7 consecutive days above 25 °C (*n* = 10; β =  −1.5; Kendall’s τ =  −0.54, *p* = 0.031, *n *= 10; pseudo-R^2^ ≈ 0.39); **b** *Ph. papatasi*, versus Julian date 7-day running mean temperature exceeding 25 °C (*n* = 10; Theil–Sen slope β = 0.64; Kendall’s τ = 0.54, *p* = 0.031, *n* = 10; pseudo-R^2^ ≈ 0.14)

Across all climate-phenology analyses, significant associations were detected for two species with thermal onset variables derived from ERA5 data using a 25 °C threshold. Median seasonal timing for *Ph. tobbi* was negatively associated with the onset of the hot season, defined as five out of 7 consecutive days above 25 °C (*n* = 10, Theil-Sen slope =  −1.5, Kendall’s τ =  − 0.54, *p* = 0.031; pseudo-R^2^ ≈ 0.39; Fig. 6a). By contrast, median seasonal timing of *Ph. papatasi* was positively associated with the onset defined by a 7-day mean temperature above 25 °C (*n* = 10, *Theil-Sen slope* = 0.64, *Kendall’s τ* = 0.54, *p* = 0.031; pseudo-R^2^ ≈ 0.14; Fig. 6b). Full model outputs are provided in the additional file, Tables S5 and S6.

## Discussion

### Human and climate impacts on vector populations

Human-induced spatial changes, including the conversion of natural habitats into anthropogenic landscapes and the creation of artificial environments that support reservoir hosts and sand fly populations, have been widely identified as key drivers of sand fly expansion and the spread of *Leishmania* worldwide [4, 15, 19, 42]. Building on previous work, the present study provides long-term, multi-species empirical evidence and shows how sand fly populations respond over time to anthropogenic habitat modification under variable climatic conditions. To our knowledge, this study is the first to document the combined long-term effects of anthropogenic environmental change and climate variability on the population dynamics of multiple sand fly species acting as vectors for three distinct *Leishmania* species [18].

The exceptionally long time series (18 years), together with relatively high sampling intensity at a fixed site and well-characterized human-driven environmental modifications in one site, provides a unique opportunity to examine changes in sand fly populations across multiple temporal scales. This contrasts with most previous studies, which have either assessed sand fly distributions in a binary manner in relation to environmental and climatic conditions at coarse spatial scales [5, 13], or focused on seasonal patterns derived from time series that are too short to capture the influence of long-term drivers [20, 21, 43].

### Species-specific responses to environmental drivers

Human-driven environmental change emerged as a dominant contextual factor shaping sand fly population dynamics in this study, resulting in species-specific and phase-dependent responses over time, whereby the effect of environmental factors changed across different stages following habitat modification rather than remaining constant over time. Habitat suitability was reshaped following land-cover modification, producing temporary increases, subsequent declines, and contrasting trajectories among species.

Changes in land use and land cover may, among other effects, influence local microclimatic conditions. For example, through the reduction of rock crevices owing to ground disturbance on the one hand, and increased humidity resulting from irrigation and shading associated with the park on the other. These changes may in turn affect both larval development and adult activity, particularly within sheltered microhabitats, such as soil cracks and rock crevices, where sand flies develop and rest [20, 43, 46].

The ability to interpret the patterns observed in the current time series is limited. First, the long-term monitoring data, by their nature, lack a “control site” free from environmental change effects. Second, the starting point of the sampling period was largely arbitrary, and data on population size prior to the disturbance are not available for comparison. Nevertheless, the results suggest that human activity affected two species (*P. sergenti* and *P. papatasi*), resulting in relatively high abundance during, and particularly in the years immediately following the change in land-cover and land-use, while this effect gradually declined over time. By contrast, the two other species, *Ph. tobbi* and *Ph. syriacus*, showed higher values after the same event.

The temporal patterns observed for *Ph. sergenti* and *Ph. papatasi* can be interpreted as a shared phase-dependent response to the land-cover active disturbance, such as soil movement, construction, and exposed substrates, which may cause an increase in their relative abundance for several seasons, followed later on by a decrease along with the process of landcover stabilization [32, 33]. An additional explanation for the pattern of the temporal response of *Ph. sergenti, which* performed a more gradual decline, maybe related to the reduction of its preferred rocky habitats, which also supports its primary reservoir—host (*P. capensis*) habitats, [15, 30, 44]. By contrast, the relatively rapid decline after the peak observed in *Ph. papatasi* is plausibly linked to anthropogenic modification, specifically to development-related disturbance. This species is known to be particularly widespread in semi-arid Mediterranean agricultural landscapes [34, 45, 46], and is therefore likely to exhibit a preference for continuously disturbed and cultivated soils.

By contrast, the two species belonging to the subgenus *Larroussius*, *Ph. tobbi* and *Ph. syriacus,* showed an initial increase following the park establishment, followed by irregular peak years thereafter, indicating a different response to the land-use change. This pattern is likely linked to increased irrigation and the expansion of perennial vegetation in the park nearby. Both species are characterized by a relatively mesic distribution and habitat preferences [13, 47], and can therefore be assumed to be highly dependent on human-mediated environmental conditions in a semi-desert setting such as the study area.

The park was located approximately 200–300 m from the sampling site, a distance that falls within the typical dispersal range reported for some sand fly species [34]. Therefore, it is plausible that the observed changes in species composition reflect local responses to environmental modifications associated with the land use changes of the park establishment and activity. However, as the study was based on a single sampling location, spatial gradients in response to distance could not be explicitly evaluated.

Conceptually, it seems that sand fly responses to human-induced change are not uniform but vary across stages, consistent with a phase-dependent disturbance response framework [48]. During active landscape changes (e.g., soil movement and construction), temporary breeding opportunities may increase abundance. As landscapes stabilize (e.g., surface sealing and managed green areas), suitability may decline for some species, leading to reduced abundance or shifts in species composition. Both processes can occur at the same location over time, so disruption does not uniformly increase or decrease sand fly populations; it reshapes habitat suitability, with different effects across species [33].

Within this context, climatic variability was then examined as an additional factor influencing seasonal dynamics and interannual variability. Despite the inherent challenge of disentangling the effects of environmental and climatic drivers on populations, our structured analysis demonstrates a partial climatic effect alongside the influence of landscape change.

All four sand fly species exhibited a seasonal pattern, characterized by absent or very low activity during the winter, a gradual increase in spring, peak activity in summer, and a decline in autumn, consistent with the common Mediterranean climatic condition. This pattern reflects the role of temperature in defining the seasonal window of activity [20, 22]. However, interannual temperature variability across multiple temporal scales was not positively correlated with sand fly abundance, suggesting that while temperature constrains seasonal activity, it does not alone explain year-to-year fluctuations in population dynamics.

Apart from the correlation observed between *Ph. sergenti* and *Ph. papatasi* in the median seasonal timing of peak population abundance (which serves as a proxy for seasonal population growth dynamics), no significant correlations were detected among species in the seasonal timing of peak activity. This lack of synchrony indicates a variability between species in seasonal dynamics and suggests nonuniform responses to temperature-related drivers across sand fly species.

Among the four sand fly species examined, only *Ph. tobbi* exhibited a response in both the timing of population increase and peak magnitude to interannual and intra-annual temperature variability. This relationship was opposite to commonly reported patterns of a positive relationship between abundance and temperature [20], reflecting sensitivity to high temperature. Years characterized by higher population peaks were associated with relatively moderate temperature increases early in the season, while earlier population growth was associated with slower rates of temperature increase. It is worth noting that, in one previous study of *Ph. tobbi* conducted in Turkey, there was one observation suggesting that climatic factors influence intra-seasonal variability, as reflected in a bimodal seasonal pattern. Specifically, a mid-summer decline in population size was observed, which may be associated with climatic constraints during a peak of heat and aridity, and may consequently affect the magnitude of seasonal peaks [22].

Taken together, these findings suggest that *Ph. tobbi* may preferentially respond to relatively moderate thermal conditions. This response may be attributed to the exceptionally hot and semi-arid nature of the study site, which lies near the climatic margin of the Mediterranean zone. It is therefore plausible that such a response would not be observed in cooler Mediterranean environments.

It should be noted that the interpretation of the historical data presented here is limited by the reliance on male-based identification, with female species competition inferred from male proportions. This approach assumes a relatively constant sex ratio across species and time. [17, 20, 47]. However, sex ratios may vary in response to environmental conditions and population dynamics. This limitation should be considered when interpreting patterns of specific species.

The interspecific variability observed in species traits, including responses to environmental change, sensitivity to interannual climatic drivers, and within-season temporal patterns, indicates divergent adaptive strategies to local environmental conditions and, likely, to distinct microhabitats. This heterogeneity poses a substantial challenge for developing effective, integrative strategies for mitigating increasing sand fly populations and reducing *Leishmania* exposure near human settlements.

## Conclusions

The study site examined here serves as a case study for four of the dominant *Leishmania* vectors in West Asia and Southern Europe. While its exceptionally arid conditions warrant caution when extrapolating these findings to more humid regions, the results from this study underscore the influence of land management and spatial planning on sand fly populations dynamics over extended time scales. Importantly, human activity did not uniformly increase or decrease sand fly abundance but altered habitat suitability over time, producing temporary increases, subsequent declines, and species-specific responses. These phase-dependent effects emphasize the limitations of generalized assumptions regarding the impact of environmental disturbance on vector populations. We therefore recommend that landscape management in peri-urban environments explicitly consider the long-term and stage-dependent consequences of habitat modification. Sustained entomological monitoring, combined with detailed documentation of land-use and management practices, is essential for detecting delayed and species-specific responses and for supporting effective leishmaniasis risk assessment in human-modified landscapes.

## Supplementary Information

Supplementary Material 1.

## Data Availability

The datasets generated and analyzed during the current study are derived from ongoing routine national vector surveillance conducted by the Medical Entomology Laboratory (Ministry of Health, Israel) and the Division of Pest Control and Pesticides (Ministry of Environmental Protection, Israel). The data are subject to institutional and regulatory restrictions and are therefore not publicly available. Relevant summary data are provided within the manuscript and supplementary materials.

## Abbreviations

CL: Cutaneous leishmaniasis
VL: Visceral leishmaniasis
NDVI: Normalized difference vegetation index
STAC: SpatioTemporal asset catalogs
IMS: Israel Meteorological Service
ERA5: The fifth generation ECMWF atmospheric reanalysis
