# Supplementary material for: Long-term monitoring of sand fly populations: temporal responses to land-use change and climatic variability
Source: Parasit Vectors. 2026 Jun 5;19:324. doi: 10.1186/s13071-026-07475-1 (PMC13459740; doi:10.1186/s13071-026-07475-1)
Supplement: Supplementary file 1 [file 13071_2026_7475_MOESM1_ESM.docx]

**Long-Term Monitoring of Sand Fly Populations: Temporal Responses to Land-Use Change and Climatic Variability - Additional file**

**Figure S1**. Temperature measurements for IMS and ERA5 sources (18:00) for 2007-2021.

**Figure S2.** A comparison between daily IMS and ERA5 temperature values.

**Figure S3.** Daily differences between IMS and ERA5 as a function of mean temperature in IMS.

**Figure S4.** Monthly bias between IMS and ERA5 evening temperature (18:00)

**Figure S5**. correlation between mean temperatures between IMS and ERA5 for 2007-2021.

**Tables**

**Table S1**. Pearson correlation coefficients between IMS- and ERA5-derived onset dates calculated using the 5-of-7 days above threshold definition. Correlations are shown for all combinations of temperature thresholds applied to each dataset. Values are presented as correlation coefficient *r*, followed by sample size (*n*) and associated *p*-value in parentheses. Significant correlations (p < 0.05) are indicated in bold.

**Table S2.** Pearson correlation coefficients between IMS- and ERA5-derived onset dates calculated using the 7 days mean threshold definition. Correlations are shown for all combinations of temperature thresholds applied to each dataset. Values are presented as correlation coefficient *r*, followed by sample size (*n*) and associated *p*-value in parentheses. Significant correlations (p < 0.05) are indicated in bold.

**Table S3.** Theil-Sen regression models for the potential effect of sampling effort (number of observations per season) for the maximal abundance (max catch), and the timing of the seasonal median and maximal abundance. Significant correlations (p < 0.05) are indicated in bold.

**Table S4.** Theil-Sen regression models between maximum sand fly abundance and climatic variables, Israel Meteorological Service data. (N = 15 years, 2007–2021). Bolded rows indicate statistically significant relationships.

**Table S5.** Theil-Sen regression models between the timing of median seasonal sand-fly’s abundance and the onset climatic variables, ERA5 data. (N = 10 years, 2005–2014). Bolded rows indicate statistically significant relationships.

**Table S6.** Theil-Sen regression models between maximum sand fly Maximum abundance timing and climatic variables, ERA5 data. (N = 10 years, 2005–2014).
